# A Lac-caninum Case

**Published:** 1892-04

**Authors:** Rufus Choate

**Affiliations:** Washington, D. C.


### A LAC-CANINUM CASE.

Rufus Choate, M. D., Washington, D. C.

J. F., a boy, aged five years, spent Wednesday evening with a company of children and got more candy than was good for him.

Thursday I was called and found a very sick boy. He was restless and no place could hold him. If he went to his mother's lap, her arms were too heavy for him; if lying down, his body pressed too heavily upon the bed. He tossed about—never at rest.

He complained of a headache as of a nail driven into his head. His face was flushed and his whole body in a red glow. I was told that he awoke in the morning with a sore throat.

Upon examining his throat I found it highly congested, and the tonsils, with fine red veins visible. On the right tonsil, about the size of the nail of the little finger, a dirty, dark patch of membrane, and the same in the back of the throat. A smaller particle of membrane on the left tonsil.

He was awakened from sleep by empty swallowing, which caused him to cry out with pain.

The back of his hands and the upper portions of his feet itched horribly, and he was constantly calling to be scratched.

His eyes were congested and full of tears.

He scratched his head vigorously.

He says his legs feel as though cold water was poured upon them.

Pulse, 136. Temperature, 101.

I called for two glasses, one about half-full of water. Into the water were placed about twenty No. 10 pellets, medicated with Lac-caninum $50^{M}$ (Swan), and for one minute I poured the medicated water from glass to glass. To the boy was given one teaspoonful (what remained was thrown out and and the glasses sent out of the room to be washed). With the same care, water medicated with Sac-lac. was prepared and the order given to let the patient have one teaspoonful of this every two hours.

Friday morning.—During the night the palms of the hands and the soles of the feet itched so fearfully that only rubbing with a hair-brush appeased somewhat its fury.

Upon examination I found his throat much less congested. The patch on the left side almost gone; no membrane in the back of the throat, and very much less on right tonsil.

The lad received me with a smile.

Pulse, 100. Temperature, 98.6.

Gave him one more teaspoonful Lac-can. and continued Sac-lac. every two hours with directions that to-morrow a report of his condition be sent to my office.

The following report was sent to me Saturday by the patient's mother :

Jack rested well all night ; and I gave him his medicine every two hours. I have just examined his throat and find the right side looking better than last night ; the left side still shows the white patch a little. Behind the palate is a bright red spot. The whole surface, of course, is still inflamed, but looks better than it did.

I am writing to tell you of the eruption that has appeared. His legs and arms, and from the small of his back down to the insteps of his feet are covered with a fine rash. I don't think I could put a pin-point between them. He is wild with the itching.

I sent more Sac-lac. Teaspoonful every two hours.

Sunday morning.—The boy, under two doses of Lac-caninum, $50^{M}$ (Swan) is doing splendidly. I prescribed more Sac-lac. and in four days, am happily through with a serious case.

